# Extracellular Matrix Oxidised by the Granulocyte Oxidants Hypochlorous and Hypobromous Acid Reduces Lung Fibroblast Adhesion and Proliferation In Vitro

**DOI:** 10.3390/cells10123351

**Published:** 2021-11-29

**Authors:** Michael Papanicolaou, Patrick He, Sandra Rutting, Alaina Ammit, Dikaia Xenaki, David van Reyk, Brian G. Oliver

**Affiliations:** 1 Faculty of Science, School of Life Sciences, University of Technology Sydney, Sydney, NSW 2007, Australia; Michael.Papanicolaou@student.uts.edu.au (M.P.); Patrick.He@student.uts.edu.au (P.H.); Alaina.Ammit@uts.edu.au (A.A.); David.VanReyk@uts.edu.au (D.v.R.); 2 Garvan Institute of Medical Research, Darlinghurst, NSW 2010, Australia; 3 Woolcock Emphysema Centre, Woolcock Institute of Medical Research, University of Sydney, Sydney, NSW 2037, Australia; 4 Woolcock Respiratory Cellular and Molecular Biology, Woolcock Institute of Medical Research, The University of Sydney, Sydney, NSW 2037, Australia; sandra.rutting@sydney.edu.au (S.R.); dia.xenaki@sydney.edu.au (D.X.)

**Keywords:** ECM, fibrosis, HOX

## Abstract

Chronic airway inflammation and oxidative stress play crucial roles in the pathogenesis of chronic inflammatory lung diseases, with airway inflammation being a key driving mechanism of oxidative stress in the lungs. Inflammatory responses in the lungs activate neutrophils and/or eosinophils, leading to the generation of hypohalous acids (HOX). These HOX oxidants can damage the extracellular matrix (ECM) structure and may influence cell–ECM interactions. The ECM of the lung provides structural, mechanical, and biochemical support for cells and determines the airway structure. One of the critical cells in chronic respiratory disease is the fibroblast. Thus, we hypothesised that primary human lung fibroblasts (PHLF) exposed to an oxidised cell-derived ECM will result in functional changes to the PHLF. Here, we show that PHLF adhesion, proliferation, and inflammatory cytokine secretion is affected by exposure to HOX-induced oxidisation of the cell-derived ECM. Furthermore, we investigated the impact on fibroblast function from the presence of haloamines in the ECM. Haloamines are chemical by-products of HOX and, like the HOX, haloamines can also modify the ECM. In conclusion, this study revealed that oxidising the cell-derived ECM might contribute to functional changes in PHLF, a key mechanism behind the pathogenesis of inflammatory lung diseases.

## 1. Introduction

Neutrophilic and/or eosinophilic infiltration of the lungs is a prominent feature of several inflammatory lung diseases, including chronic obstructive pulmonary disease (COPD), asthma, and asthma–COPD overlap syndrome (ACOS) [1,2,3,4,5,6,7,8]. These inflammatory cells produce hypohalous acids (HOX), hypochlorous acid (HOCl), and hypobromous acid (HOBr), the products of reactions between hydrogen peroxide and their respective halides, catalysed by the haem peroxidases myeloperoxidase (MPO) and eosinophil peroxidase (EPO) [9]. Established as key features of the neutrophil and eosinophil antimicrobial armoury, the products of MPO and EPO activity have been shown also to modify tissue and cell components [9,10,11,12,13,14,15,16,17,18,19,20]. Such modifications could lead to alterations in the functionality of the targeted tissue.

Tissue homeostasis refers to the collection of processes that ensure the optimal functioning of cells within a tissue, including the tissue microenvironment and architecture [21]. Fibroblasts play a central role in tissue homeostasis. The interaction between the extracellular matrix (ECM) and the fibroblast is bidirectional: the fibroblasts can modulate the ECM composition and the ECM modulates fibroblast function [22,23].

The potential for oxidative modification of ECM by oxidants, including MPO- and EPO-derived oxidants, has been previously investigated [24,25,26,27,28,29,30]

This study investigated the impact upon human lung fibroblast function of in vitro exposure to ECM, where the ECM was previously exposed to either HOCl or HOBr at physiological concentrations.

## 2. Materials and Methods

### 2.1. Primary Cell Isolation

Primary human lung fibroblasts (PHLF) were isolated from the parenchyma of explanted or resected lungs from hospitals in Sydney. The mean age of volunteers was 57, all of whom were diagnosed with thoracic malignancies (26%) and/or IPF (16%), COPD 32%, or other diseases/no diagnosis was available (26%). A 1 cm^3^ piece of lung parenchyma was finely chopped into approximately 1 mm^3^ pieces and washed with sterile Hank’s buffered salt solution (HBSS). The tissue pieces were seeded in 75 cm^2^ flasks (minimum 10 pieces per flask, Thermo Fisher Scientific, Waltham, MA, USA) and maintained in 5% FBS/antibiotics/DMEM at 37 °C and 5% CO_2_. Fibroblasts identified by morphology, growth patterns and lack of alpha smooth muscle actin staining were confluent by 2–4 weeks post-dissection and were subsequently passaged. All experiments were performed using PHLF between passage 2 and 6.

### 2.2. Cell-Derived ECM

To generate the cell-derived ECM, BEAS-2B human bronchus epithelial cells were utilised, purchased from the American Type Culture Collection (ATCC CRL-9609). Cells were seeded at a density of 1 × 105 cells/mL in 10% FBS/antibiotics/DMEM for 72 h at 37 °C and 5% CO_2_ in 96-well and 24-well plates (Thermo Fisher Scientific. Waltham, MA, USA). The cells were subsequently washed twice with phosphate-buffered saline (PBS), then decellularized with 16 mM NH4OH, leaving an intact ECM. Cellular debris was then removed with two washes of PBS. PBS was added to each well, then plates were sealed with parafilm and stored at −20 °C. Plates were thawed out overnight at 4 °C prior to use.

### 2.3. Preparation of HOCl Stocks

HOCl (purchased from Bacto Laboratories Pty Ltd (Sydney, NSW, Australia) catalogue # ST044-500M) concentration of stock solution was determined spectrophotometrically at 292 nm, with a molar extinction coefficient ℇ292 of 350 M^−1^ cm^−1^ [1]. A 340 mM working stock solution of HOCl was prepared in Milli-Q water and serially diluted in PBS (340 µM, 170 µM, and 85 µM final concentrations) prior to use. Concentrations were chosen in accordance with prior estimates of physiological HOCl production by activated neutrophils [2].

### 2.4. Preparation of HOBr Stocks

HOBr was freshly made up for each experiment. Firstly, 45 mM NaBr (from Sigma-Aldrich, Castle Hill, NSW, Australia) and 40 mM HOCl solutions were prepared in milli-Q water. A ratio of 1.125:1 NaBr to HOCl was reacted for 60 min at room temperature (RT) to produce HOBr. Following its production, HOBr concentration was determined spectrophotometrically (λ = 329 nm, ε329 = 332L mol^−1^ cm^−1^, at pH 10–12) [31]. HOBr was then serially diluted to working concentrations of 340 µM, 170 µM, and 85 µM.

### 2.5. HOCl and HOBr Treatment of Cell-Derived ECM

BEAS-2B-derived ECM plates were washed twice with PBS before being incubated with or without HOCl/HOBr (85 µM, 170 µM, or 340 µM) for 30 min at room temperature (RT). HOCl/HOBr was removed with two washes of PBS. Where indicated in Figures 7 and 8, plates were incubated with or without methionine (1 mM) for 10 min at 37 °C and 5% CO_2_ to remove haloamines that may also contribute to modifications of the ECM [3]. To block subsequent non-specific cell binding, the plates were blocked with sterile 1% (*w*/*v*) bovine serum albumin (BSA) in PBS for 1 h at RT. Plates were then washed two additional times in PBS to remove residual BSA.

### 2.6. Cell Attachment Assay

Attachment assays were performed). Briefly, PHLFs were seeded at 1 × 105 cells/mL in 0.1% (*v*/*v*) FBS/antibiotics/DMEM for 2 h at 37 °C and 5% CO_2_ upon BEAS-2B-derived ECM in 96-well plates. The plates were washed three times with PBS to remove any unbound cells. Cells were fixed with 4% (*v*/*v*) formaldehyde/PBS for 5 min at RT and after an additional PBS wash, stained for 5 min with 0.01% (*w*/*v*) Coomassie Brilliant Blue R-250/20% (*v*/*v*) methanol/10% (*v*/*v*) acetic acid. Plates were washed once with PBS, then dye was released with 1% (*w*/*v*) SDS/milli-Q water before absorbance was measured at 595 nm using a SpectraMax M2 microplate reader (Molecular Devices, Sunnyvale, CA, USA) [4].

### 2.7. Proliferation Assay

PHLF were seeded at 6 × 10^4^ cells/mL in 5% FBS/antibiotics/DMEM for 24 h, 48 h, 72 h, and 96 h at 37 °C and 5% CO_2_ upon BEAS-2B-derived ECM in 24-well plates. Adherent PHLF were detached with 0.05% (*w*/*v*) trypsin/HBSS for 3 min at 37 °C, before inactivating the trypsin with FBS. Manual cell counts were performed every 24 h using a 0.4% (*w*/*v*) trypan blue exclusion assay to count viable cells.

### 2.8. ELISA

Supernatant of samples assessed for proliferation were used to detect secreted interleukin (IL)-6 and IL-8. Supernatants were stored at −20 °C until further analysis. Determination of IL-6 and IL-8 levels was performed by ELISA, according to the manufacturer’s instructions (BD Biosciences Pharmingen, San Diego, CA, USA).

### 2.9. Data Analysis and Statistics

All results are presented as the mean ± standard error of mean (SEM) from at least 5 independent experiments. Statistical significance was determined by one-way analysis of variance (ANOVA) with Dunnett’s multiple comparisons test, two-way ANOVA with Tukey multiple comparisons test, or two-tailed paired Student’s *t*-test using GraphPad Prism 9.0 (GraphPad Prism Inc., La Jolla, CA, USA), shown by *p* < 0.05 (* *p* < 0.05, ** *p* < 0.01).

## 3. Results

### 3.1. Characterization of Oxidized Collagen

**Figure 1:**
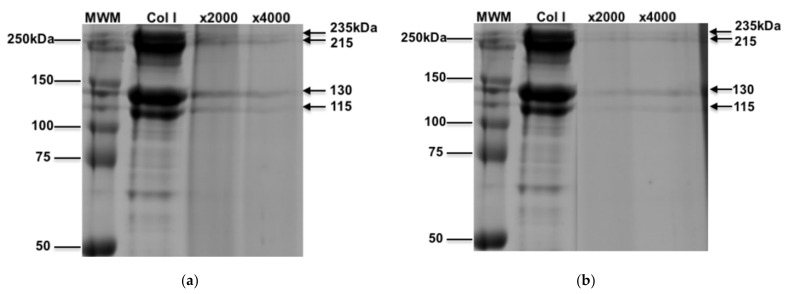
Coomassie blue staining of HOCl-oxidised type I collagen SDS-PAGE gel. Type I collagen (20 µg/lane) was electrophoresed on an 8–10% (*v*/*v*) SDS-PAGE gel under reducing conditions prior to staining with Coomassie Brilliant Blue R-250. The four arrows represent the protein bands (235, 215, 130, 115kDa) present in the type I collagen solution. Type I collagen was incubated with (**a**) HOCl or (**b**) HOBr at molar ratios of 1:2000 and 1:4000 collagen to HOCl for 30 min before loading. The gel presented is a representative gel of 3 independent experiments.

We assessed the physical nature of oxidized collagen by SDS-PAGE gel electrophoresis and Coomassie brilliant blue R-250 staining. Collagen was exposed to HOCl or HOBr at molar ratios of 1:2000 (×2000) and 1:4000 (×4000), which is reflective of 85 µM and 170 µM of HOCl or HOBr, respectively. When unoxidized, collagen exists as four predominant bands at 235, 215, 130, and 115 kDa, as shown in Figure 1, whereas the oxidised collagen staining of the bands were fainter in addition to smearing, reflecting a 3D conformational change such as removal of cross linkages or cleavage of collagen, in a concentration-dependent effect.

### 3.2. Altered Fibroblast Function Following HOCl-Induced Oxidation of the Cell-Derived ECM

**Figure 2:**
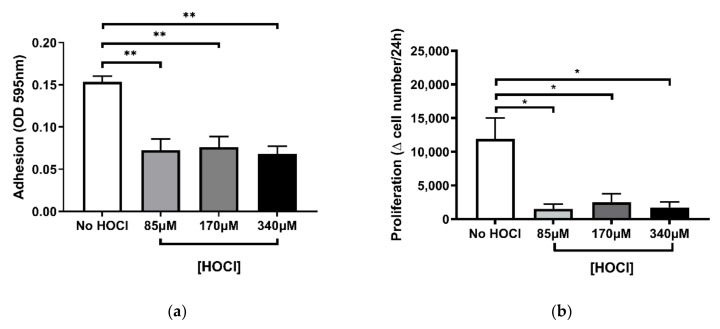
Cell-derived ECM oxidised by HOCl reduces the capacity of fibroblasts to adhere and proliferate. BEAS-2B-derived ECM was oxidised with (or without) HOCl concentrations of 85 µM, 170 µM, and 340 µM for 30 min, then washed and seeded with PHLF at 1 × 10⁵ cells/mL in 0.1% FBS DMEM for 2 h. Attachment was assessed by Coomassie brilliant blue staining (**a**) and 6.0 × 10⁴ cells/mL in 5% FBS DMEM for 96 h, while proliferation was measured by manual cell counts (**b**). *n* = 5. ** *p* < 0.01, * *p* < 0.05, significance was compared against No HOCl control; one-way ANOVA with Dunnett’s multiple comparison test was performed.

Damage to the ECM is known to negatively regulate cell adhesion, proliferation, and signalling [5]. However, the consequences of oxidative damage to the ECM on PHLF function remain unclear [5]. To examine these functional changes, we first assessed the ability of PHLF to attach to the oxidised BEAS-2B-derived ECM, referred to as cell-derived ECM (Figure 2a). The results showed that there was a significant reduction in PHLF attachment across all concentrations (85 µM, 170 µM, 340 µM) of HOCl-induced oxidation of the ECM (*p* < 0.01; Figure 2a) when compared to the untreated (No HOCl) control. However, there were no differences in PHLF response between the HOCl concentrations. To determine the rate of PHLF proliferation, we observed the change in fibroblast count every 24 h (Figure 2b). To compare the proliferation with the outcomes of the attachment assay, the same range of HOCl concentrations (85 µM, 170 µM, 340 µM) were used. Similar to the PHLF response in the attachment assay, HOCl suppressed PHLF proliferation at all concentrations (*p* < 0.05; Figure 2b) compared to control.

**Figure 3:**
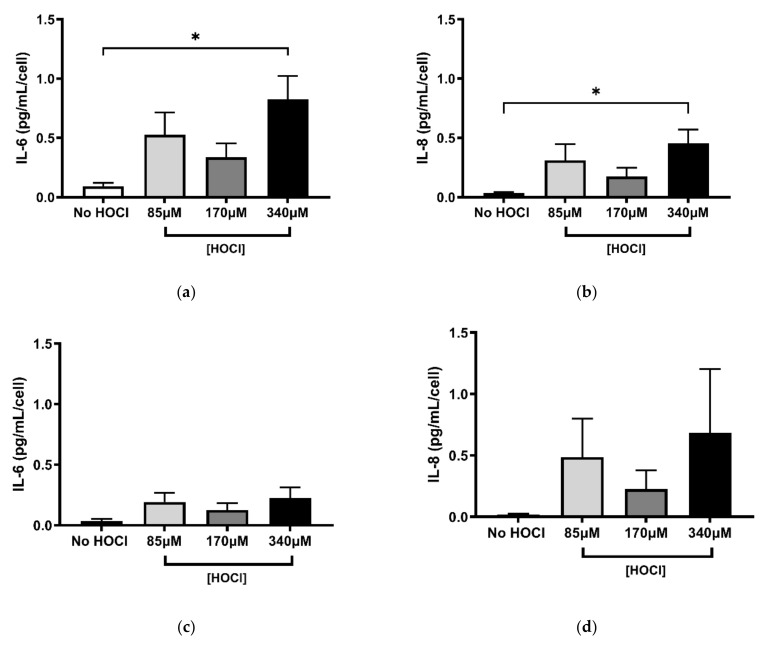
Effects of HOCl-oxidized cell-derived ECM on fibroblasts’ secretion of inflammatory cytokines. Fibroblasts were grown on BEAS-2B-derived ECM oxidised with (or without) HOCl (85 µM, 170 µM, and 340 µM) and supernatants were collected after 24 h (**a**,**b**) and 96 h (**c**,**d**) to measure the concentrations of IL-6 A, C and IL-8 B, D by ELISA. Results were obtained as picograms per microlitre per cell (normalised against the cell counts at 24 h and 96 h post-seeding) and are expressed as mean ± SEM. A, B, C, D *n* = 5. * *p* < 0.05, significance was compared against No HOCl control; one-way ANOVA with Dunnett’s multiple comparison test was performed.

In contrast to the HOCl-induced reduction in both PHLF attachment and proliferation, PHLF seeded on the oxidised ECM only significantly increased IL-6 and IL-8 at the highest HOCl concentration, 24 h post-seeding (*p* < 0.05; Figure 3a,b). Meanwhile, at 96 h, no change to the release of IL-6 or IL-8 was observed (Figure 3c,d). These data suggest that HOCl-induced oxidation of the ECM is capable of dramatically changing PHLF function in vitro.

### 3.3. HOBr-Induced Oxidation of the Cell-Derived ECM on Fibroblast Function Compared to HOCl Treatment

To assess the differences in response between HOBr- and HOCl-induced oxidation of the ECM on PHLF function, we further analysed the functional changes of PHLF when exposed to the ECM, which has been oxidised with HOBr.

**Figure 4:**
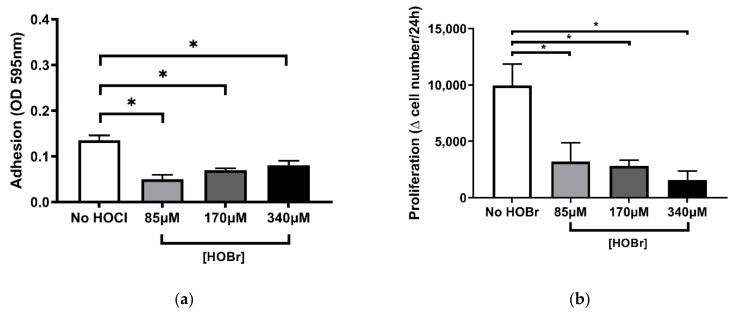
HOBr-induced oxidation of cell-derived ECM showed similar effects on fibroblast adhesion and proliferation compared to HOCl. BEAS-2B-derived ECM was oxidised with (or without) HOBr concentrations of 85 µM, 170 µM, and 340 µM and seeded with PHLF for 2 h (**a**) and for 96 h (**b**). (**a**) Attachment was assessed by Coomassie brilliant blue staining; (**b**) manual cell counts. Results are averages of the ∆ (changes) in cell number per 24 h and expressed as mean ± SEM. A, B *n* = 5. * *p* < 0.05, significance was compared against No HOBr control; one-way ANOVA with Dunnett’s multiple comparison test was performed.

Attachment and proliferations assays for HOBr were performed under the same concentrations (85 µM, 170 µM, 340 µM) as shown previously for HOCl (Figure 4a,b). Results showed a significant reduction in PHLF adhesion, consistent for all HOBr concentrations as compared to the untreated (No HOBr) control (*p* < 0.05; Figure 4a). A decrease in PHLF proliferation was expressed for all HOBr concentrations, showing a negative relationship between HOBr concentration and proliferation rate (Figure 4b). Response of PHLF to attachment and proliferation on the ECM oxidised with HOBr has thus far been consistent with prior outcomes with HOCl (Figure 2).

**Figure 5:**
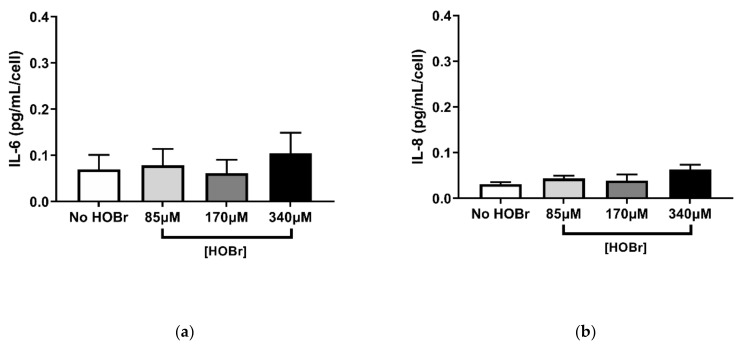

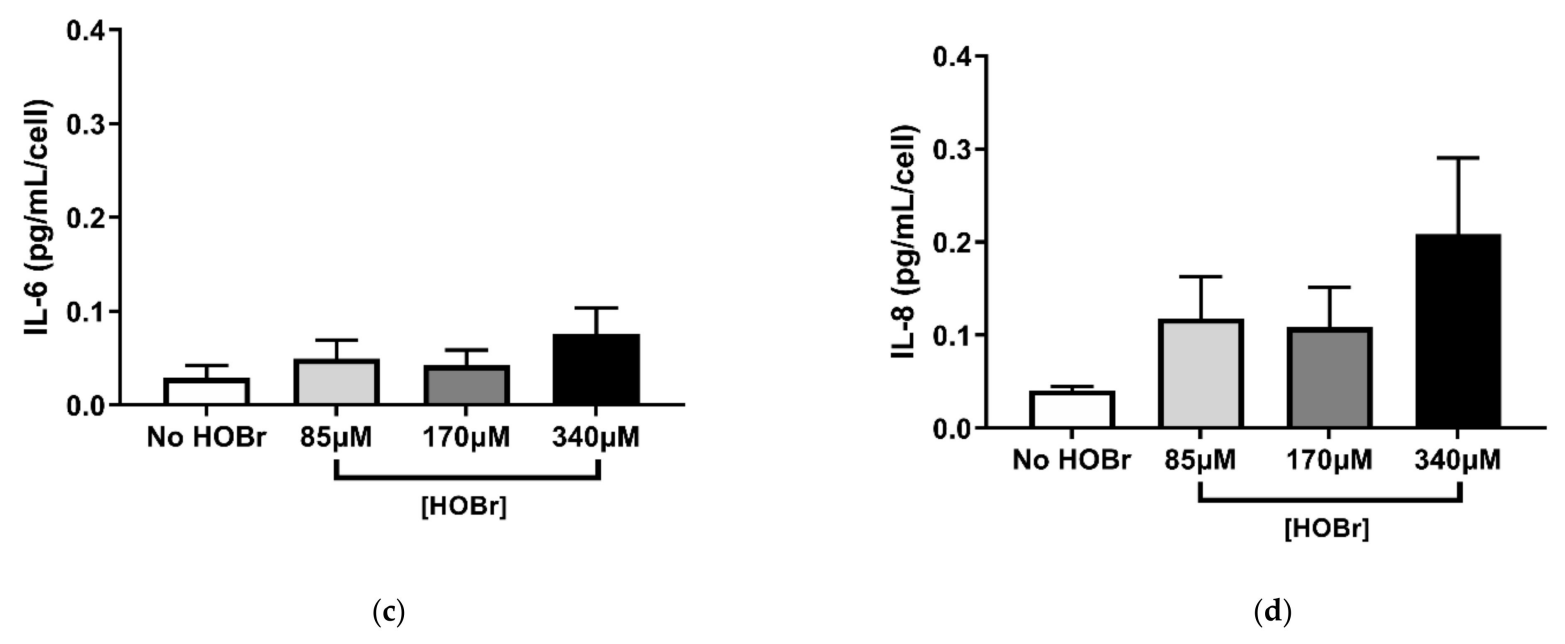
Effects of HOBr-oxidised ECM on fibroblasts’ secretion of inflammatory cytokines. BEAS-2B-derived ECM was oxidised with (or without) HOCl concentrations of 85 µM, 170 µM, and 340 µM for 30 min and seeded with PHLF. Supernatants were collected after 24 h (**a,b**) and 96 h (**c**,**d**) to measure the concentrations of IL-6 (**a**,**c**) and IL-8 (**b**,**d**) by ELISA. Results were picograms per microlitre per cell (normalised against the cell counts at 24 h and 96 h post-seeding) and expressed as mean ± SEM. (**a–d**) *n* = 5.

In contrast to what was observed for HOCl, HOBr-induced oxidation of the ECM did not produce increased IL-6 and IL-8 secretion at either 24 h or 96 h post-seeding when compared to the control (Figure 5). These results indicate that HOBr treatment elicited a weaker response on fibroblast function when compared to HOCl.

### 3.4. Fibroblast Morphology When Grown on Oxidized ECM

**Figure 6:**
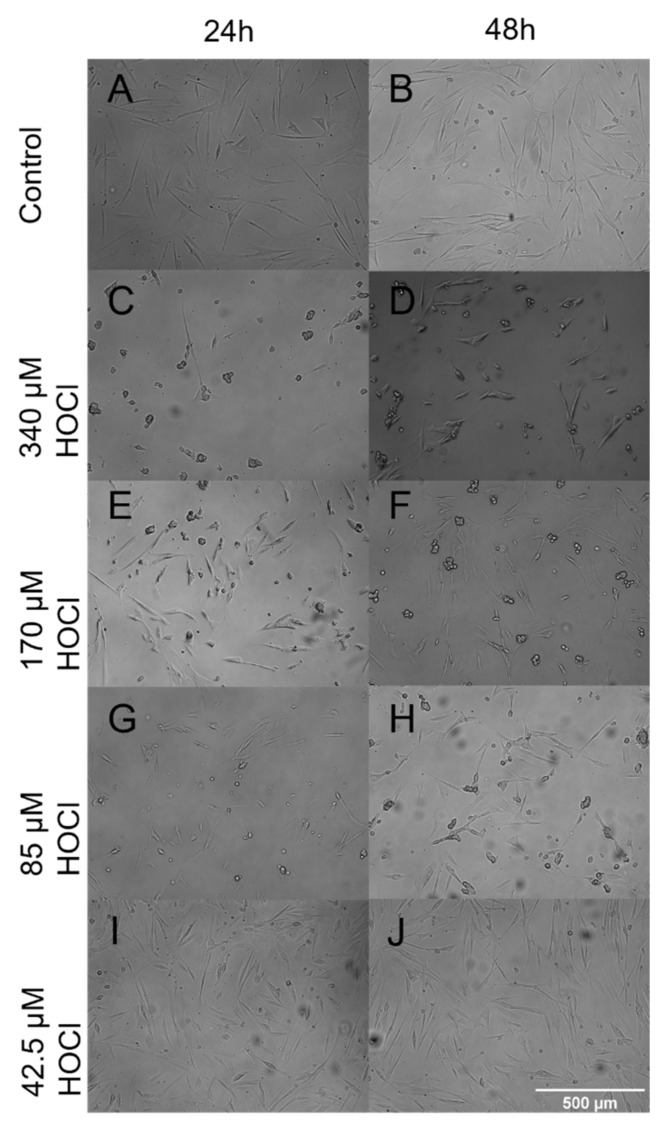
Bright-field microscopy. Bright-field microscopy images of the changes in cell morphology of primary human lung fibroblasts post-exposure to HOCl at 24 h and 48 h timepoints. (**A**–**J**) were viewed at 10× magnification using the time-lapse microscope. Images are representative of *n* = 5.

Given the changes in attachment and proliferation, we next sought to investigate if any changes to cell morphology were caused by growth on oxidized ECM. As can be seen in Figure 6, PHLF grown on ECM oxidized by 340 µM HOCl were stellate and less elongated compared to the untreated ECM. However, the morphology changes were subtle when grown on ECM oxidized by lower concentrations of HOCl.

### 3.5. Involvement of Haloamines in the Modification of ECM on Fibroblast Function

HOCl and HOBr reacts with amines to generate the haloamines (chloramines and bromamines, respectively) [6,7,8]. Similar to their parent HOX oxidants, chloramines and bromamines can oxidize the ECM and alter cellular function [6,7,8]. To clarify the previously observed functional changes in PHLF were the result of HOCl- or HOBr-induced modification of the ECM, methionine was utilised in the quenching of chloramines and bromamines. Previously tested functional assays were re-examined, however, in addition to incubating the cell-derived ECM for 30 min with HOCl or HOBr (85 µM, 170 µM, 340 µM), ECM was incubated for 10 min with methionine (1 mM).

**Figure 7:**
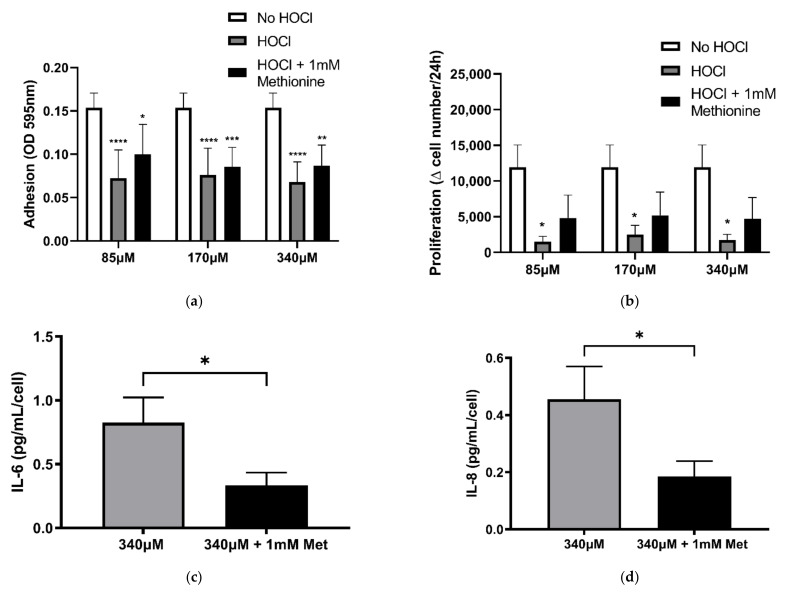
Comparison of the effects of chloramine quenched against HOCl-induced oxidation of BEAS-2B-derived ECM on fibroblast function. BEAS-2B derived ECM was oxidised with (or without) HOCl concentrations of 85 µM, 170 µM, and 340 µM for 30 min, and was then incubated for 10 min with or without methionine (1 mM) to quench chloramine. PHLF were seeded onto these matrices for 2 h (**a**) and 96 h (**b**–**d**). (**a**) Attachment was assessed by Coomassie brilliant blue staining; (**b**) manual cell counts. IL-6 (**c**) and IL-8 (**d**) release at 24 h was measured by ELISA. *n* = 5 for all. **** *p* < 0.0001, *** *p* < 0.001, ** *p* < 0.01, * *p* < 0.05, significance was compared against No HOCl; two-way ANOVA with Tukey’s multiple comparison test was performed. (**c**,**d**) *n* = 5. * *p* < 0.05, significance was compared against 340 µM HOCl; 2-tailed paired Student *t* test was performed.

Shown in Figure 7a,b, PHLF adhesion and proliferation were not reduced with or without methionine treatment on the ECM oxidised with HOCl. The results suggest that suppression of PHLF attachment and proliferation from HOCl induced oxidation of the cell-derived ECM previously observed in Figure 2, independent of the ECM’s chloramine modification. However, treatment with HOCl (340 µM) and methionine induced a decrease in IL-6 and IL-8 at 24 h (Figure 7c,d; *p* < 0.05) when compared with 340 µM HOCl alone, indicating that chloramines were associated with the increase in IL-6 and IL-8 secretion by PHLF 24 h post-seeding on cell-derived ECM oxidised with 340 µM of HOCl (Figure 3a,b).

**Figure 8:**
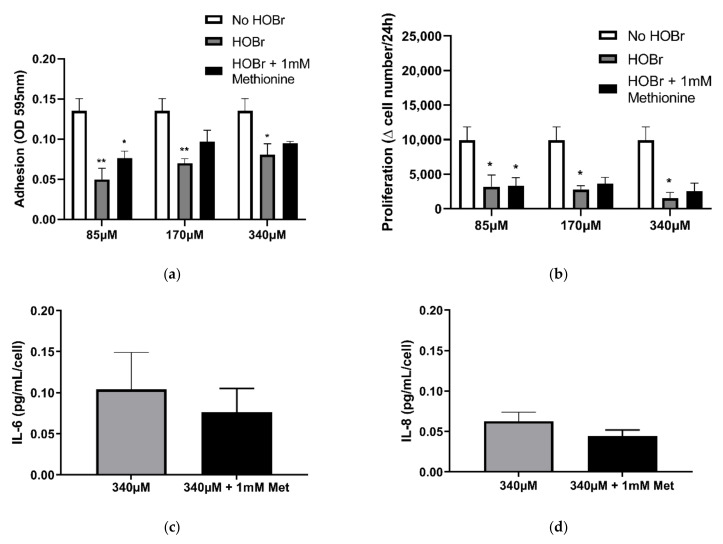
Comparison of the effects of bromamine quenched against HOBr-induced oxidation of BEAS-2B-derived ECM on fibroblast function. BEAS-2B-derived ECM was oxidised with (or without) HOBr concentrations of 85 µM, 170 µM, and 340 µM and then incubated for 10 min with or without methionine (1 mM) to quench chloramine, followed by seeding with PHLF. (**a**) Attachment was assessed by Coomassie brilliant blue staining. (**b**) Manual cell counts at 24 h. Results are averages of the ∆ (changes) in cell number per 24 h and expressed as mean ± SEM. IL-6 (**c**) and IL-8 (**d**) release measured by ELISA. Results were obtained as picograms per microlitre per cell (normalised against the cell counts at 24 h post-seeding) and are expressed as mean ± SEM. (**a**,**b**) *n* = 5. ** *p* < 0.01, * *p* < 0.05, significance was compared against No HOBr; two-way ANOVA with Tukey’s multiple comparison test was performed. (**c**,**d**) *n* = 5. * *p* < 0.05, significance was compared against 340 µM HOBr; 2-tailed paired Student *t* test was performed.

Methionine treatment of bromamines, showed no difference in PHLF attachment and proliferation between HOBr-induced oxidation of cell-derived ECM with or without methionine (Figure 8a,b). These findings confirm that bromamines were not involved in reducing PHLF attachment and proliferation observed in Figure 4. Together, these results indicate that haloamines in the ECM were not involved in the functional changes to PHLF attachment and proliferation due to HOCl- and HOBr-induced oxidation of the cell-derived ECM. However, chloramines may have contributed to the increased IL-6 and IL-8 release in PHLF shown by HOCl treatment (Figure 7c,d). This was indicated when we compared the HOCl-oxidised ECM with and without methionine treatment—we observed a significantly lower secretion in IL-6 and IL-8 after the removal of chloramines with methionine treatment.

## 4. Discussion

This study aimed to address the functional changes in PHLF when exposed to an oxidised ECM. There were three major findings. Firstly, we demonstrated that oxidising the cell-derived ECM with the hypohalous acid, HOCl, suppressed the adhesion and proliferation of PHLF. PHLF secretions of inflammatory cytokines IL-6 and IL-8 were also increased when the cell-derived ECM was exposed to HOCl (340 µM) at 24 h post-seeding. Secondly, oxidising the cell-derived ECM using a second hypohalous acid HOBr, similar to HOCl, was found to suppress PHLF adhesion and proliferation. Conversely, HOBr did not affect the inflammatory cytokines, with IL-6 and IL-8 levels shown to have increased with HOCl. Thirdly, the presence of chloramines in the ECM as a result of oxidising the BEAS-2B ECM with HOCl was involved with increasing IL-6 and IL-8 release by PHLF.

The ECM is an intricate network of structural and functional proteins, consisting of fibrous proteins, glycoproteins, and proteoglycans [9,10]. It plays a crucial role in providing a scaffold for the surrounding cells [11,12,13]. Bound cells are connected to the ECM by transmembrane receptors known as integrins, allowing the ECM to regulate the essential cellular functions and behaviour [11,12,13]. These include cell survival, adhesion, proliferation, signalling, migration, and differentiation to apoptotic cell death [11,12]. However, the structure and composition of the ECM are tissue-specific and also vary depending on the specific cell secreting the ECM [14,15,16]. In the lungs, primarily the epithelial cells and resident fibroblasts are responsible for ECM production [15]. Lung epithelial and endothelial cells deposit fine layers of ECM, known as the basement membrane and interstitial ECM secreted by fibroblasts [15,17]. Hence, in this study, lung bronchial epithelial BEAS-2B cells were utilised to produce a layer of ECM, which was in turn exposed to MPO-derived HOX oxidants, HOCl and HOBr.

In inflammatory lung diseases, such as COPD, asthma, and ACOS, HOX oxidants produced from activated neutrophils and eosinophils damage the ECM of the lungs [18,19]. It is well established that oxidative stress in the lungs can alter ECM structure and function [5,19,20]. However, the consequences that oxidative damage to the ECM has on the cells in the lung it regulates during cell–ECM interaction remain to be fully elucidated [5,19,20]. Hence, we investigated the effect of an oxidised ECM on lung fibroblast function.

In the present study, we found that PHLF seeded on HOX-oxidised cell-derived ECM reduced its ability to attach onto the ECM and proliferate (Figure 1 and Figure 3). These results, however, were not in complete agreement to a similar study, where Cai et al., investigate the structural changes in human coronary artery smooth muscle cells from exposure to HOCl-oxidised smooth muscle cell-derived ECM [21]. Similar to our findings, which demonstrated that reduction in PHLF attachment to HOX induced oxidation of the ECM (Figure 1 and Figure 3), Cai et al. showed reduced human coronary artery smooth muscle cell adhesion to an oxidised ECM [21]. However, contrary to our results, which showed a reduction in PHLF proliferation, Cai et al., observed a significant increase in human coronary artery smooth muscle cell proliferation [21]. It has been reported that HOX-induced modification of the ECM can be linked with increased fibroblast growth factor 2 and β3 integrin interaction, with HOCl mediation inducing transforming growth factor beta 1 (TGF-β1) expression [22,23]. Hence, it is speculated that the pro-proliferative activity of the HCASMCs may have been due to increased growth factor signalling from the ECM in response to HOX stimulation [21,24]. While there is evidence of increased TGF-β1 expression in the basement membrane of patients with COPD, there are conflicting reports of TGF-β1 expression being reduced in patients with stage 2 COPD, suggesting further research into the differences in growth factor expression between different cell types is needed [25,26,27]. Therefore, the observed decrease in PHLF proliferation with both HOX oxidants on the cell-derived ECM may be the function of tissue location and cell-specific response.

Airway inflammation occurring in inflammatory lung diseases is led by the release of pro-inflammatory cytokines by activated neutrophils and eosinophils [28,29]. In an oxidised ECM, these inflammatory cytokine genes become upregulated in response to the HOX modification of the ECM structure [21]. However, in this study, we demonstrated that only at the highest HOCl concentration did we observe a significant increase in IL-6 and IL-8 at 24 h post-seeding. In contrast, HOBr did not affect IL-6 and IL-8 levels. This can be explained by the different chemical compositions between the two HOX oxidants—HOCl has a higher redox potential, making it a more potent oxidant than HOBr [30]. This was observed throughout the PHLF functional assays, with HOCl-induced oxidation of the ECM having a more pronounced effect on fibroblast function across PHLF attachment, proliferation, and IL-6 and IL-8 secretion. However, it does not eliminate the possibility of higher cell death with HOBr treatment, resulting in lower cytokine secretion observed with HOBr treatment.

To validate that the haloamines, chloramine and bromamine, were not involved in alteration of PHLF function as a result of modifying the cell-derived ECM, methionine was used to remove the haloamines [3,32,33]. We have revealed that chloramine, but not bromamine, contributed to the observed increase in PHLF secretion of IL-6 and IL-8 at 24 h post-seeding (Figure 2a,b). Thus, in the present study, we showed that PHLF function is altered when exposed to an oxidised cell-derived ECM in vitro.

## 5. Conclusions

In conclusion, the present study demonstrates the novel finding that the ECM’s role in providing scaffolding for cells can be altered by exposure to oxidants such as HOX in inflammatory lung diseases. We have shown that in a cell-derived ECM, HOCl and HOBr can modify the ECM, having an impact on the fibroblast cells it interacts with. Fibroblast exposure to HOCl-induced oxidation of the cell-derived ECM resulted in reduced adhesion, suppressed proliferation, and increased inflammatory cytokine secretion. Similar results were shown for HOBr treatment, except for inflammatory cytokine levels remaining unchanged. Fibroblasts are fundamental in the maintenance of tissue integrity through homeostatic processes [34]. The oxidative damage to the fibroblasts can lead to possible indications of aberrant tissue homeostasis, triggering tissue remodelling of the small airways [35,36,37]. These data increase our understanding of the mechanisms behind how oxidative stress in lung ECM influences cell–ECM interactions, opening promising therapeutic targets for prevention and treatment of oxidative stress in inflammatory lung diseases.
